# Supplementary material for: Reduction of forebrain interneurons alters cellular organization and cortical neurogenesis in a mouse model of X-linked intellectual disability
Source: bioRxiv. 2026 Sep 10:2026.09.08.749487. Preprint. [Version 1] doi: 10.64898/2026.09.08.749487 (PMC13622630; doi:10.64898/2026.09.08.749487)
Supplement: Supplementary file 1 [file NIHPP2026.09.08.749487V1-supplement-1.pdf]

bioRxiv preprint doi: https://doi.org/10.64898/2026.09.08.749487; this version posted September 10, 2026. The copyright holder for this preprint (which was not certified by peer review) is the author/funder. This article is a US Government work. It is not subject to copyright under 17 USC 105 and is also made available for use under a CC0 license.

Figure S1

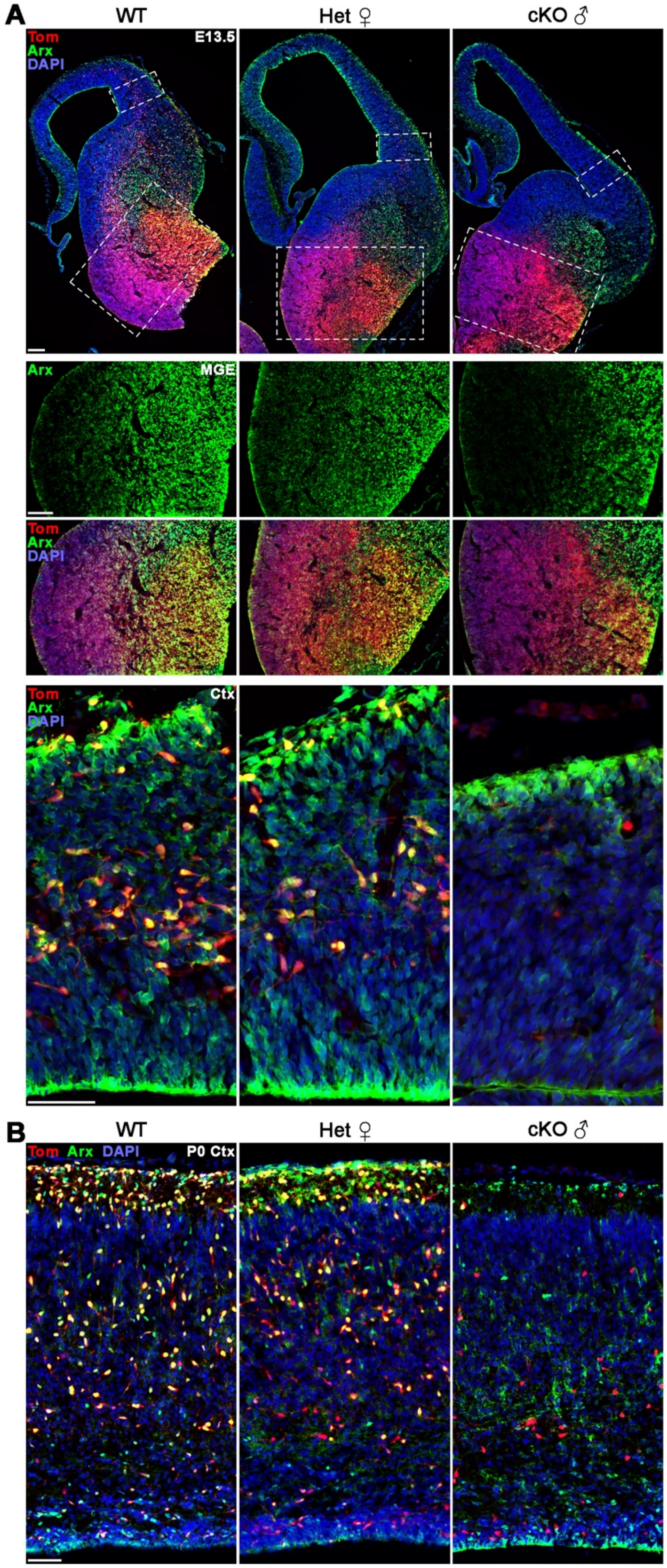

bioRxiv preprint doi: https://doi.org/10.64898/2026.09.08.749487; this version posted September 10, 2026. The copyright holder for this preprint (which was not certified by peer review) is the author/funder. This article is a US Government work. It is not subject to copyright under 17 USC 105 and is also made available for use under a CC0 license.

Figure S1. Loss of Arx protein in MGE-derived cells in cKO male mice. A. Arx protein is reduced in the MGE of E13.5 Het female and cKO male mice. Arx is expressed in migratory MGE-derived interneurons in the cortex of E13.5 WT and Het female mice, but lost in cKO male mice. White dotted boxes in top row indicate higher magnification images of MGE and lateral cortex in lower images. Scale bars = 100 μm for hemisphere and MGE, 50 μm for cortex. **B.** Tom+ MGE-derived cortical interneurons express Arx protein in P0 WT and Het female mice, but these cells are Arx-negative in cKO male mice. Scale bar = 50 μm.

bioRxiv preprint doi: https://doi.org/10.64898/2026.09.08.749487; this version posted September 10, 2026. The copyright holder for this preprint (which was not certified by peer review) is the author/funder. This article is a US Government work. It is not subject to copyright under 17 USC 105 and is also made available for use under a CC0 license.

1243 **Figure S2**

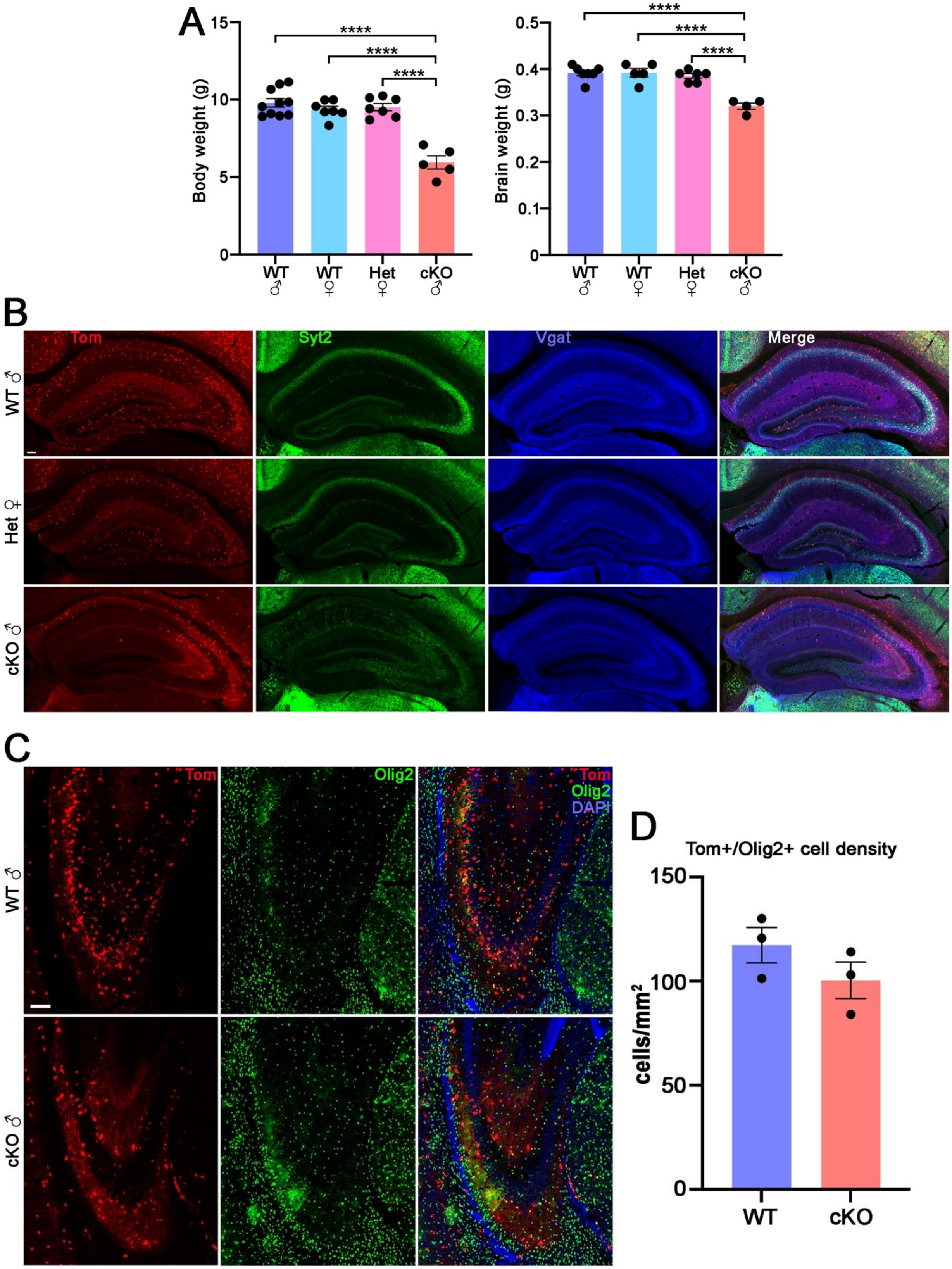

1244

bioRxiv preprint doi: https://doi.org/10.64898/2026.09.08.749487; this version posted September 10, 2026. The copyright holder for this preprint (which was not certified by peer review) is the author/funder. This article is a US Government work. It is not subject to copyright under 17 USC 105 and is also made available for use under a CC0 license.

Figure S2. Decreased body weight, brain weight and synaptotagmin-2 expression in Arx mutant mice. A. Quantification of body weight (left) and brain weight (right) from P21 WT, Het and cKO mice. n = 10 WT male, 7 WT female, 7 Het female and 5 cKO male for body weight; n = 7 WT male, 5 WT female, 6 Het female and 4 cKO male for brain weight. **B.** Reduction of synaptotagmin-2 (Syt2) in the hippocampus of P21 Arx mutant mice. **C-D.** Images highlighting Tom+/Olig2+ oligodendrocytes in CA3 in P21 male WT and cKO mice (C), and quantification of these cells (D). n = 3 for each genotype. Ordinary one-way ANOVA followed by Tukey's multiple comparison tests (A) and Welch's 2-tailed t-test (D) were used: **** = $p \leq .0001$. Scale bar = 100 μm.

bioRxiv preprint doi: https://doi.org/10.64898/2026.09.08.749487; this version posted September 10, 2026. The copyright holder for this preprint (which was not certified by peer review) is the author/funder. This article is a US Government work. It is not subject to copyright under 17 USC 105 and is also made available for use under a CC0 license.

1254

Figure S3

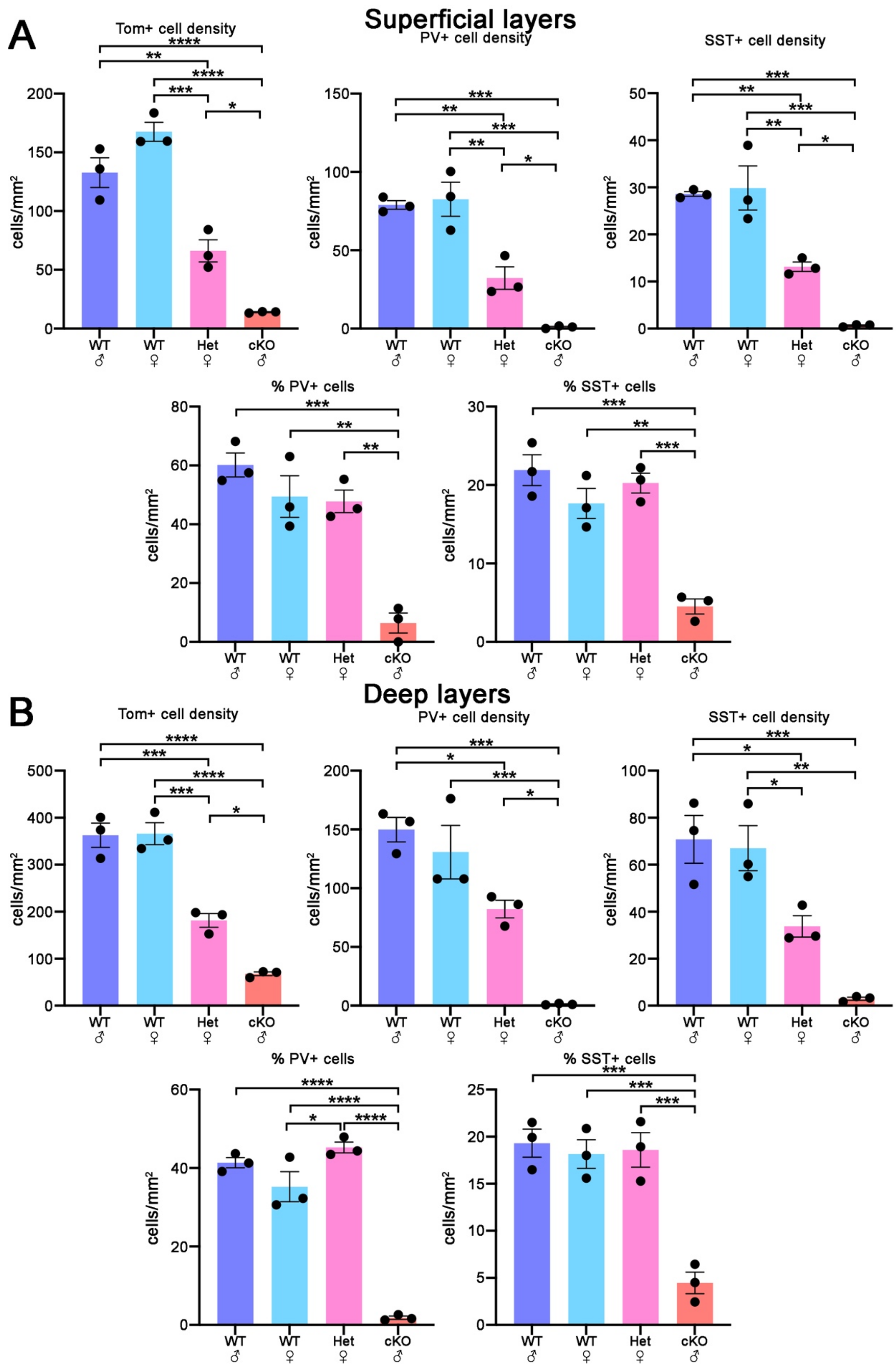

1255

bioRxiv preprint doi: https://doi.org/10.64898/2026.09.08.749487; this version posted September 10, 2026. The copyright holder for this preprint (which was not certified by peer review) is the author/funder. This article is a US Government work. It is not subject to copyright under 17 USC 105 and is also made available for use under a CC0 license.

Figure S3. Decreased MGE-derived cortical interneurons in the superficial and deep layers of Arx mutant mice. A-B. Graphs depicting the density of Tom+, PV+ and SST+ cells (top) and the percent of Tom+ cells expressing PV or SST (bottom) in the superficial layers I-III (A) and deep layers IV-VI (B) of P21 WT, Het and cKO mice. n = 3 for each condition. All stats are one-way ANOVA followed by Tukey’s multiple comparison tests: * = $p \leq .05$, ** = $p \leq .01$, *** = $p \leq .001$, **** = $p \leq .0001$.

bioRxiv preprint doi: https://doi.org/10.64898/2026.09.08.749487; this version posted September 10, 2026. The copyright holder for this preprint (which was not certified by peer review) is the author/funder. This article is a US Government work. It is not subject to copyright under 17 USC 105 and is also made available for use under a CC0 license.

1262

Figure S4

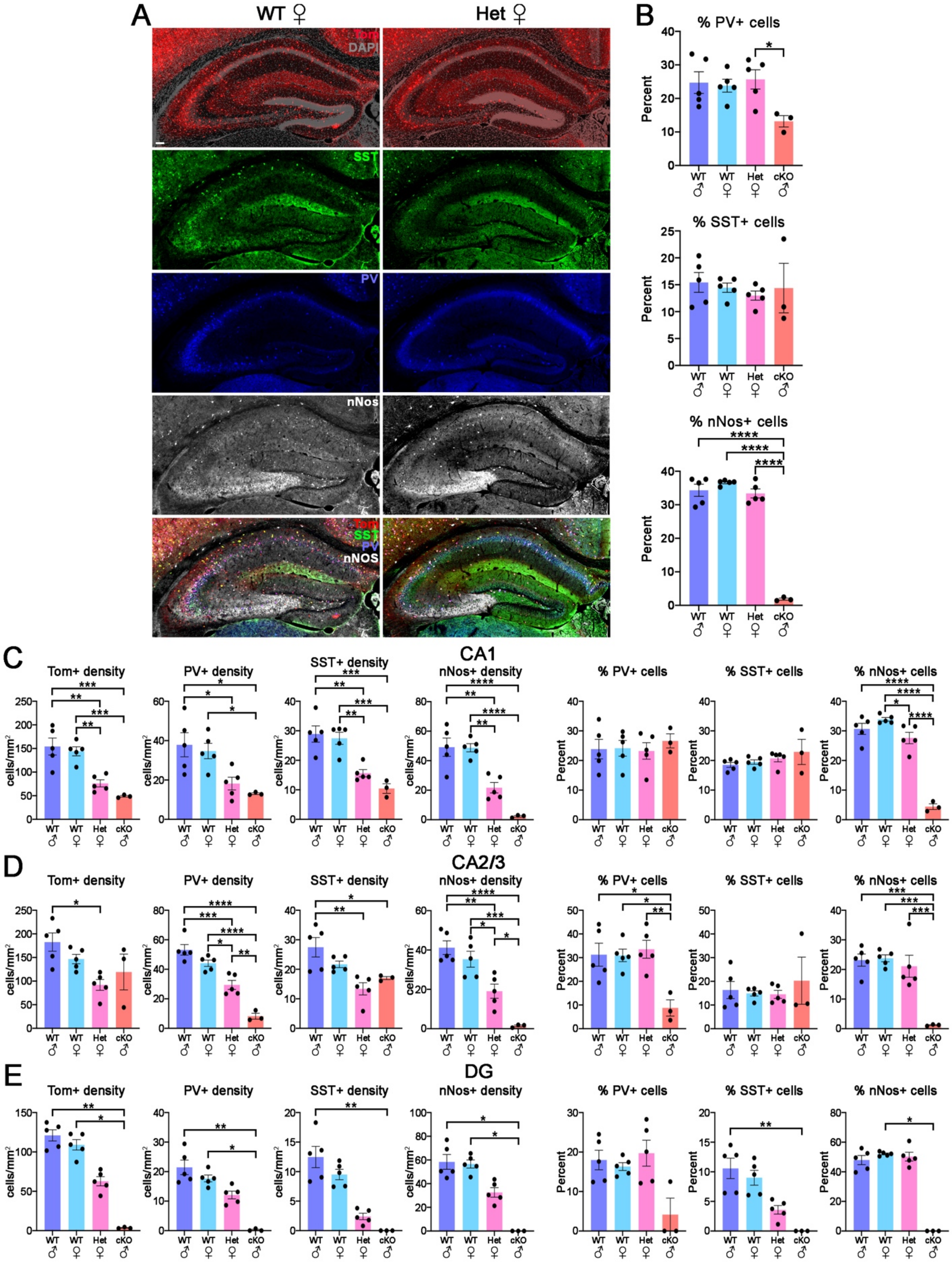

1263

bioRxiv preprint doi: https://doi.org/10.64898/2026.09.08.749487; this version posted September 10, 2026. The copyright holder for this preprint (which was not certified by peer review) is the author/funder. This article is a US Government work. It is not subject to copyright under 17 USC 105 and is also made available for use under a CC0 license.

Figure S4. Decreased MGE-derived hippocampus interneurons in Arx mutant mice. A. P21 WT and Het female hippocampus mice stained for PV, SST and nNos. Scale bar = 100 µm. **B.** Percent of hippocampal Tom+ cells expressing PV, SST or nNos in the hippocampus. **C-E.** Quantification of cell density of Tom+ PV+, SST+ and nNos+ cells (left) and percent of Tom+ cells expressing PV, SST or nNos (right) in the CA1 (C), CA2-3 (D) and DG (E). n = 5 WT male, 5 WT female, 5 Het female and 3 cKO male mice. Ordinary one-way ANOVA followed by Tukey's multiple comparison tests or Holm-Sidak's multiple comparisons test (B, C, D) and Kruskal-Wallis test followed by Dunn's multiple comparisons test (E) based on normal distribution were used: * = $p \leq .05$, ** = $p \leq .01$, *** = $p \leq .001$, **** = $p \leq .0001$.

bioRxiv preprint doi: https://doi.org/10.64898/2026.09.08.749487; this version posted September 10, 2026. The copyright holder for this preprint (which was not certified by peer review) is the author/funder. This article is a US Government work. It is not subject to copyright under 17 USC 105 and is also made available for use under a CC0 license.

Figure S5

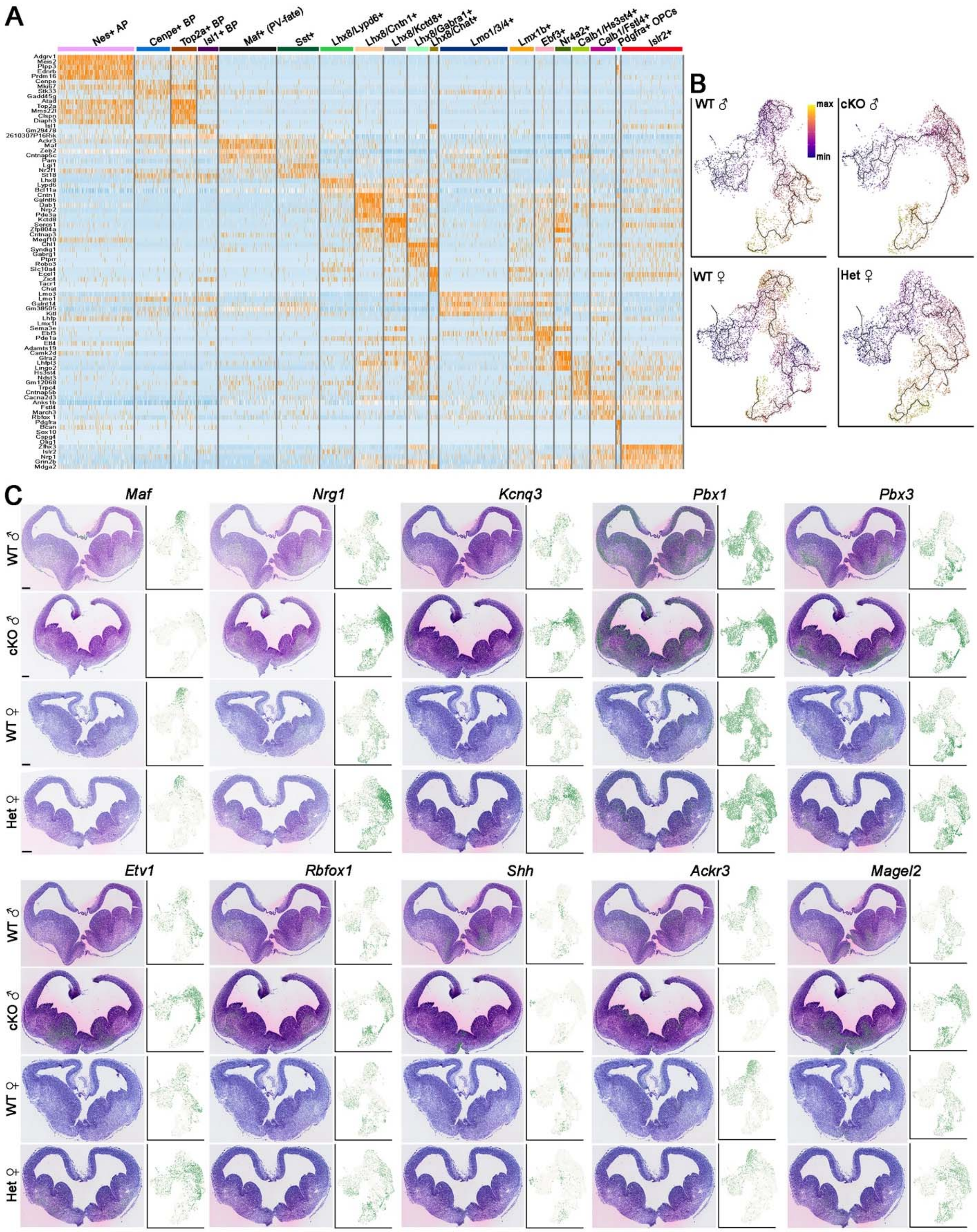

bioRxiv preprint doi: https://doi.org/10.64898/2026.09.08.749487; this version posted September 10, 2026. The copyright holder for this preprint (which was not certified by peer review) is the author/funder. This article is a US Government work. It is not subject to copyright under 17 USC 105 and is also made available for use under a CC0 license.

Figure S5. Genetically defined E13.5 cell types and spatial gene expression patterns. A. Heatmap depicting genes enriched in cell clusters of E13.5 MGE from snRNA-seq datasets. **B.** Integrated RNA and ATAC UMAP plots of E13.5 MGE cells showing pseudotime developmental trajectory using Monocle 3. **C.** mRNA expression of DEGs in E13.5 brains visualized in spatial transcriptomics data (left) and UMAP plots from snMultiome data (right). Scale bar = 200 μm.

bioRxiv preprint doi: https://doi.org/10.64898/2026.09.08.749487; this version posted September 10, 2026. The copyright holder for this preprint (which was not certified by peer review) is the author/funder. This article is a US Government work. It is not subject to copyright under 17 USC 105 and is also made available for use under a CC0 license.

Figure S6

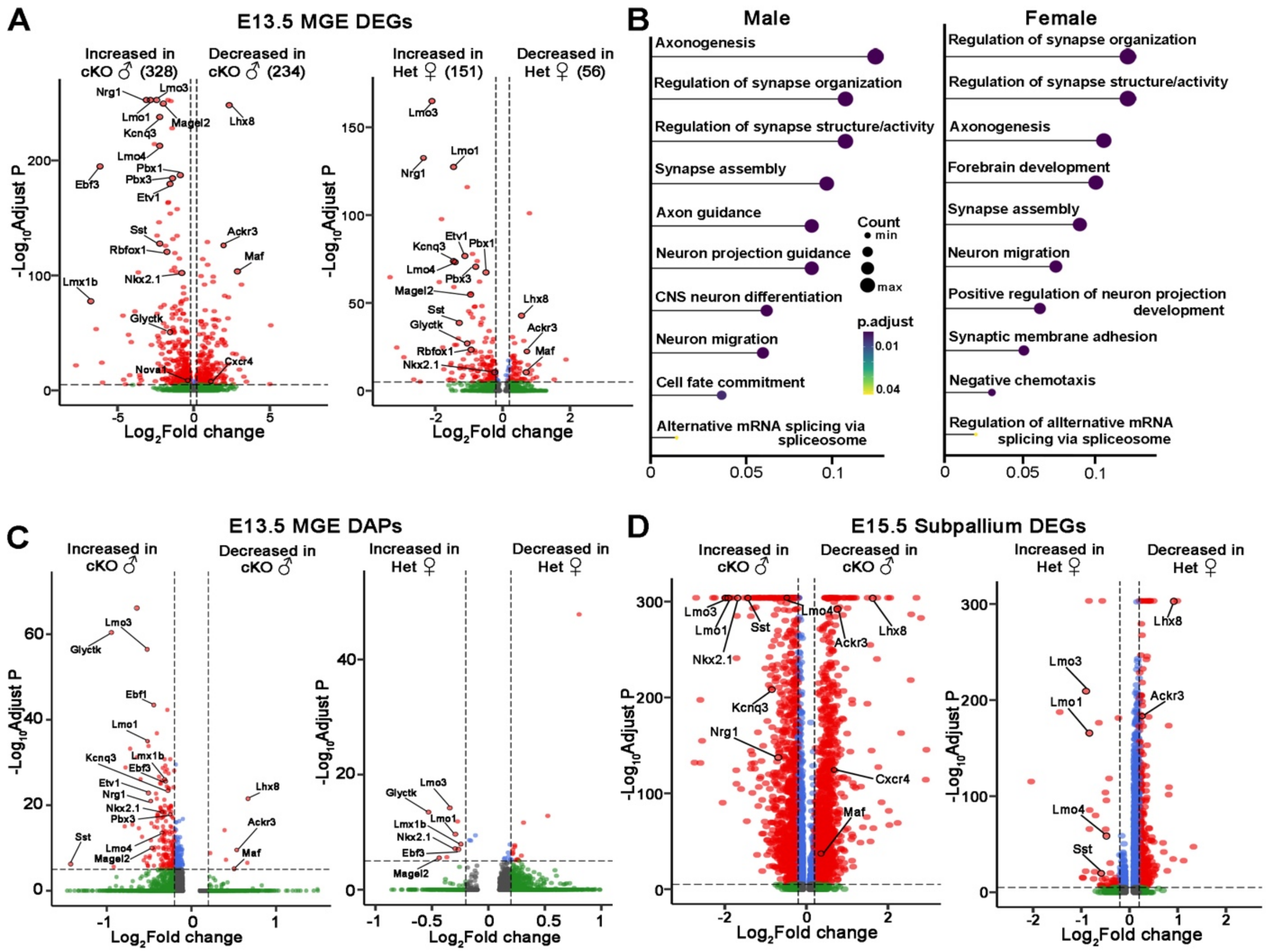

Figure S6. Differentially expression genes and differentially accessible peaks in embryonic Arx mutant brains. A. Volcano plot depicting DEGs from snRNA data in E13.5 cKO male (left) and Het female (right) MGE compared to WT. **B.** clusterProfiler GO enrichment top hits of biological processes for DEGs of E13.5 MGE in male (left) and female (right) mice. **C.** Volcano plots depicting differentially accessible peaks from snATAC data in E13.5 cKO (left) and Het (right) MGE compared to WT. **D.** Volcano plot depicting DEGs from spatial transcriptomics data in E15.5 cKO (left) and Het (right) subpallium compared to WT. Two-sided Wilcoxon rank sum test with Bonferroni correction (A, C, D) and one-sided Fisher's exact with BH correction (B) were used.

bioRxiv preprint doi: https://doi.org/10.64898/2026.09.08.749487; this version posted September 10, 2026. The copyright holder for this preprint (which was not certified by peer review) is the author/funder. This article is a US Government work. It is not subject to copyright under 17 USC 105 and is also made available for use under a CC0 license.

Figure S7

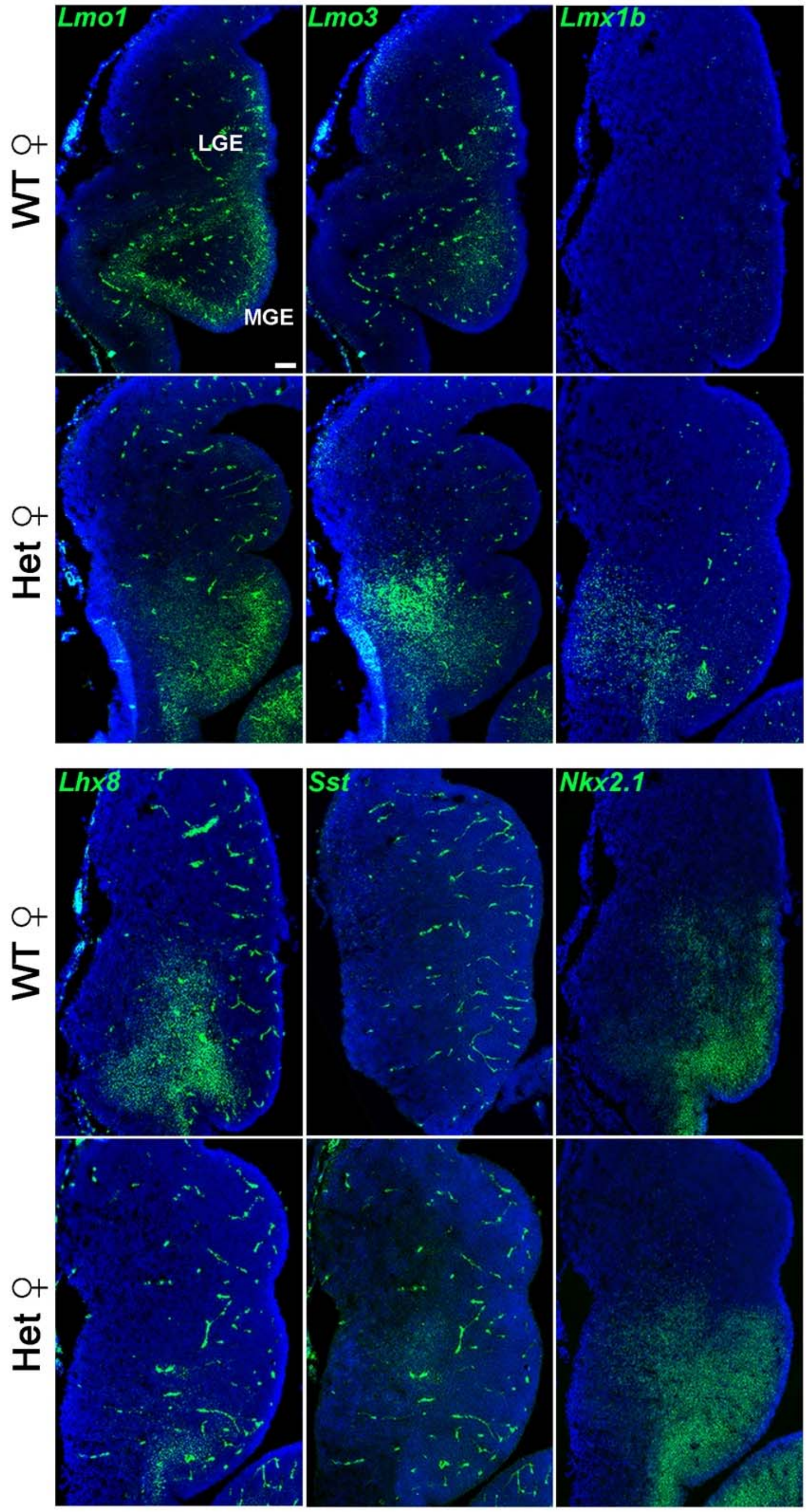

Figure S7. FISH of DEGs in E13.5 WT and Het female MGE. FISH confirmed an increase of *Lmo1, Lmo3, Lmx1b, Sst* and *Nkx2-1,* and a reduction of *Lhx8* in E13.5 female Het MGE compared to WT. Scale bar = 100 μm.

bioRxiv preprint doi: https://doi.org/10.64898/2026.09.08.749487; this version posted September 10, 2026. The copyright holder for this preprint (which was not certified by peer review) is the author/funder. This article is a US Government work. It is not subject to copyright under 17 USC 105 and is also made available for use under a CC0 license.

Figure S8

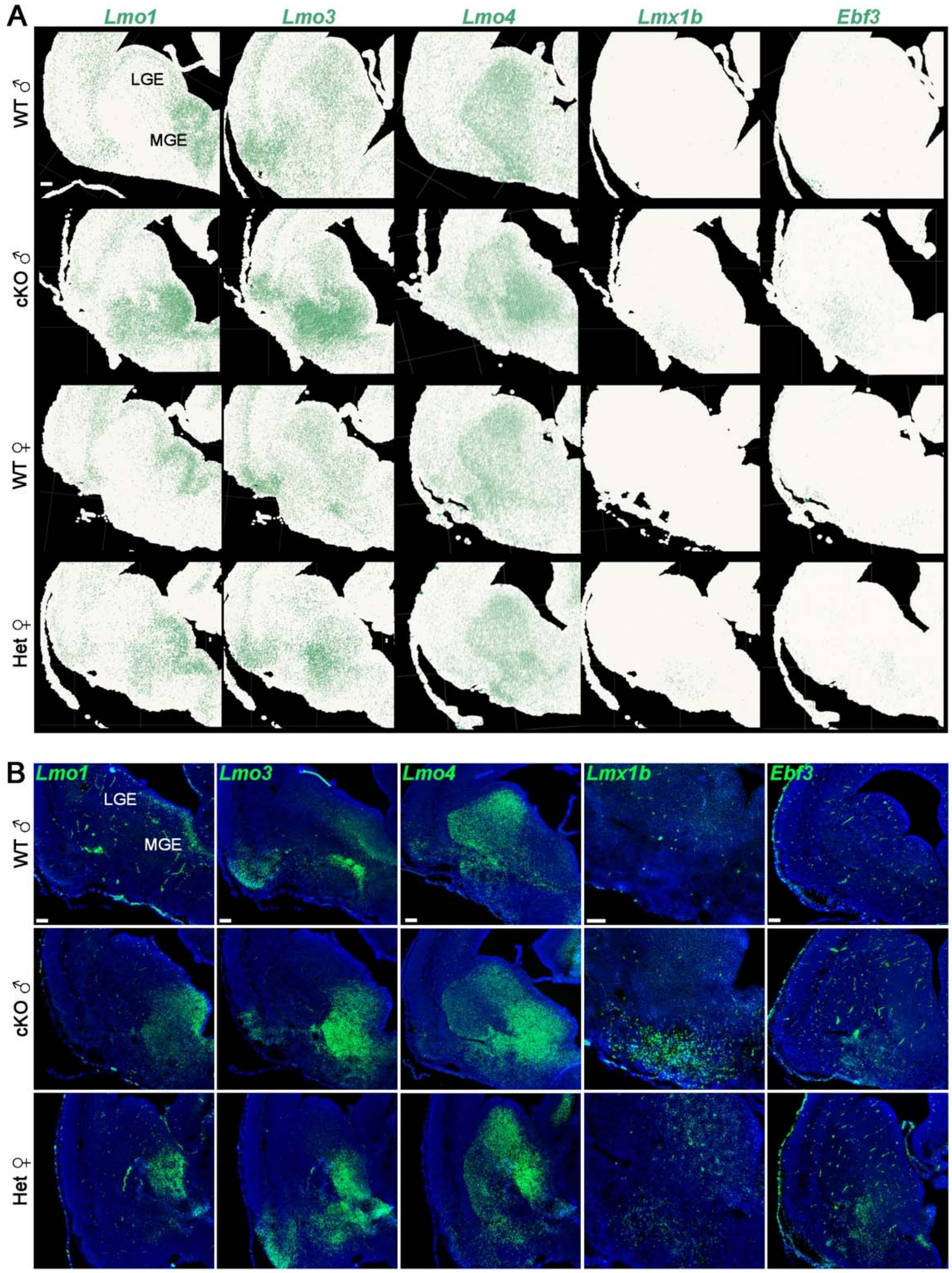

Figure S8. Spatial distribution of upregulated DEGs in E15.5 Arx mutant mice. A-B. Spatial distribution of transcripts in the E15.5 mouse brain from spatial transcriptomics data (A) and FISH visualization (B) for *Lmo1, Lmo3, Lmo4, Lmx1b* and *Ebf3.* Scale bar = 100 μm.

bioRxiv preprint doi: https://doi.org/10.64898/2026.09.08.749487; this version posted September 10, 2026. The copyright holder for this preprint (which was not certified by peer review) is the author/funder. This article is a US Government work. It is not subject to copyright under 17 USC 105 and is also made available for use under a CC0 license.

Figure S9

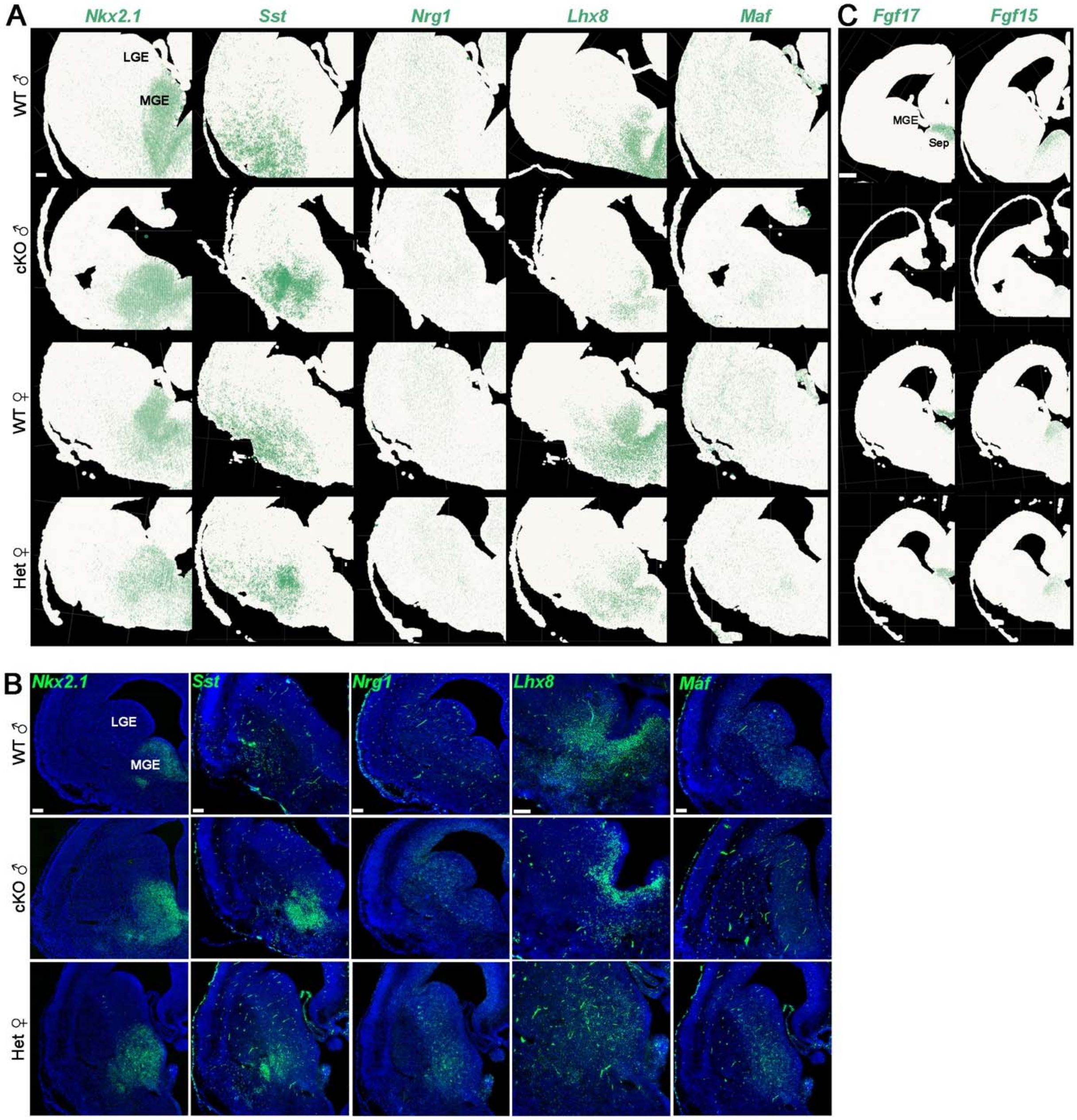

Figure S9. Spatial distribution of DEGs in E15.5 subpallium. A-B. Spatial distribution of transcripts in the E15.5 mouse brain from spatial transcriptomics data (A) and FISH visualization (B) for *Nkx2.1, Sst, Nrg1, Lhx8* and *Maf.* Scale bar = 100 µm. **C.** Spatial distribution of *Fgf17* and *Fgf15* in the E15.5 mouse brain from spatial transcriptomics data. Scale bar = 250 µm.

bioRxiv preprint doi: https://doi.org/10.64898/2026.09.08.749487; this version posted September 10, 2026. The copyright holder for this preprint (which was not certified by peer review) is the author/funder. This article is a US Government work. It is not subject to copyright under 17 USC 105 and is also made available for use under a CC0 license.

Figure S10

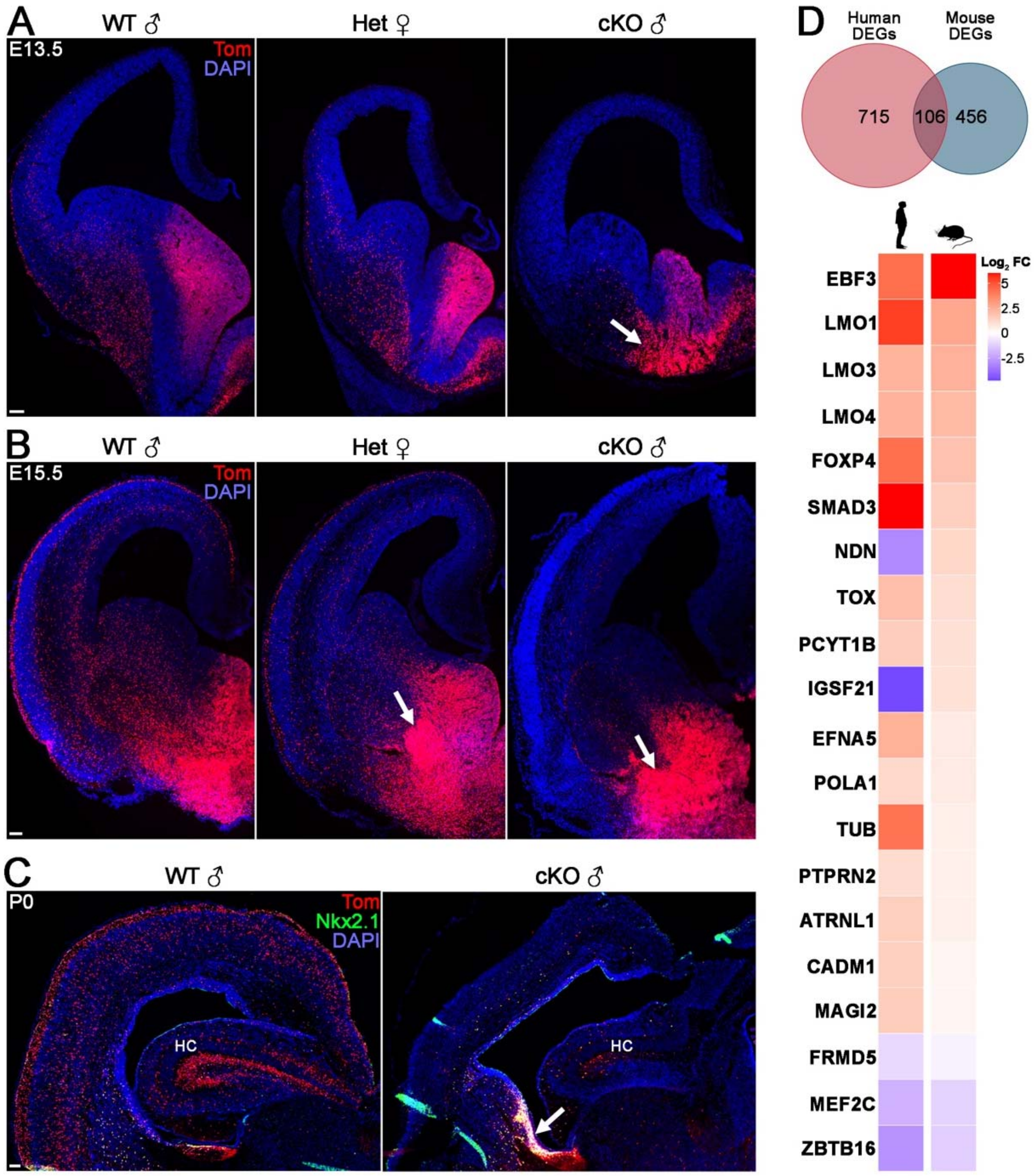

Figure S10. Migration defect from MGE-derived interneurons in Arx mutant mice. A-C. Tom+ MGE-derived interneurons get stuck in the MGE mantle in E13.5 (A) and E15.5 (B) Arx cKO mice, and Tom+ cells remain confined to the ventral forebrain periventricular zone at P0 (C). **D.** Venn diagram (top) and heatmap (bottom) showing the top hits of shared dysregulated genes upon Arx loss in both human neural progenitor cultures and E13.5 mouse MGE. Scale bar = 100 µm.

bioRxiv preprint doi: https://doi.org/10.64898/2026.09.08.749487; this version posted September 10, 2026. The copyright holder for this preprint (which was not certified by peer review) is the author/funder. This article is a US Government work. It is not subject to copyright under 17 USC 105 and is also made available for use under a CC0 license.

Figure S11

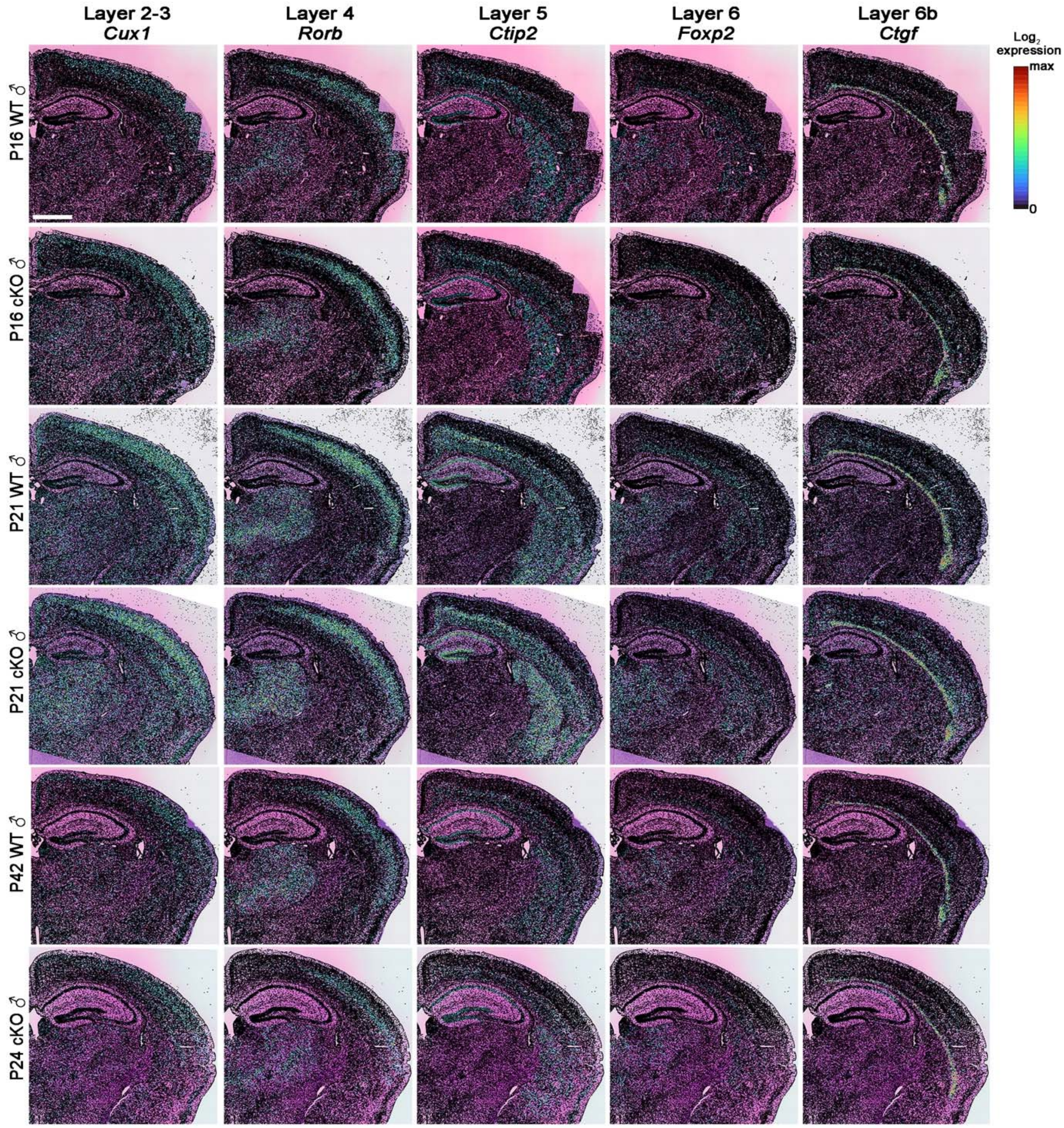

Figure S11. Spatial distribution of cortical layer markers in WT and cKO male postnatal brains. Spatial distribution of mRNA in P16, P21 and P42 WT and cKO male mice for *Cux1* (layer II-III), *Rorb* (layer IV), *Ctip2* (layer V), *Foxp2* (layer VI), and *Ctgf* (layer VIb) visualized in cell segmented spatial transcriptomics data. Scale bar = 1 mm.

bioRxiv preprint doi: https://doi.org/10.64898/2026.09.08.749487; this version posted September 10, 2026. The copyright holder for this preprint (which was not certified by peer review) is the author/funder. This article is a US Government work. It is not subject to copyright under 17 USC 105 and is also made available for use under a CC0 license.

Figure S12

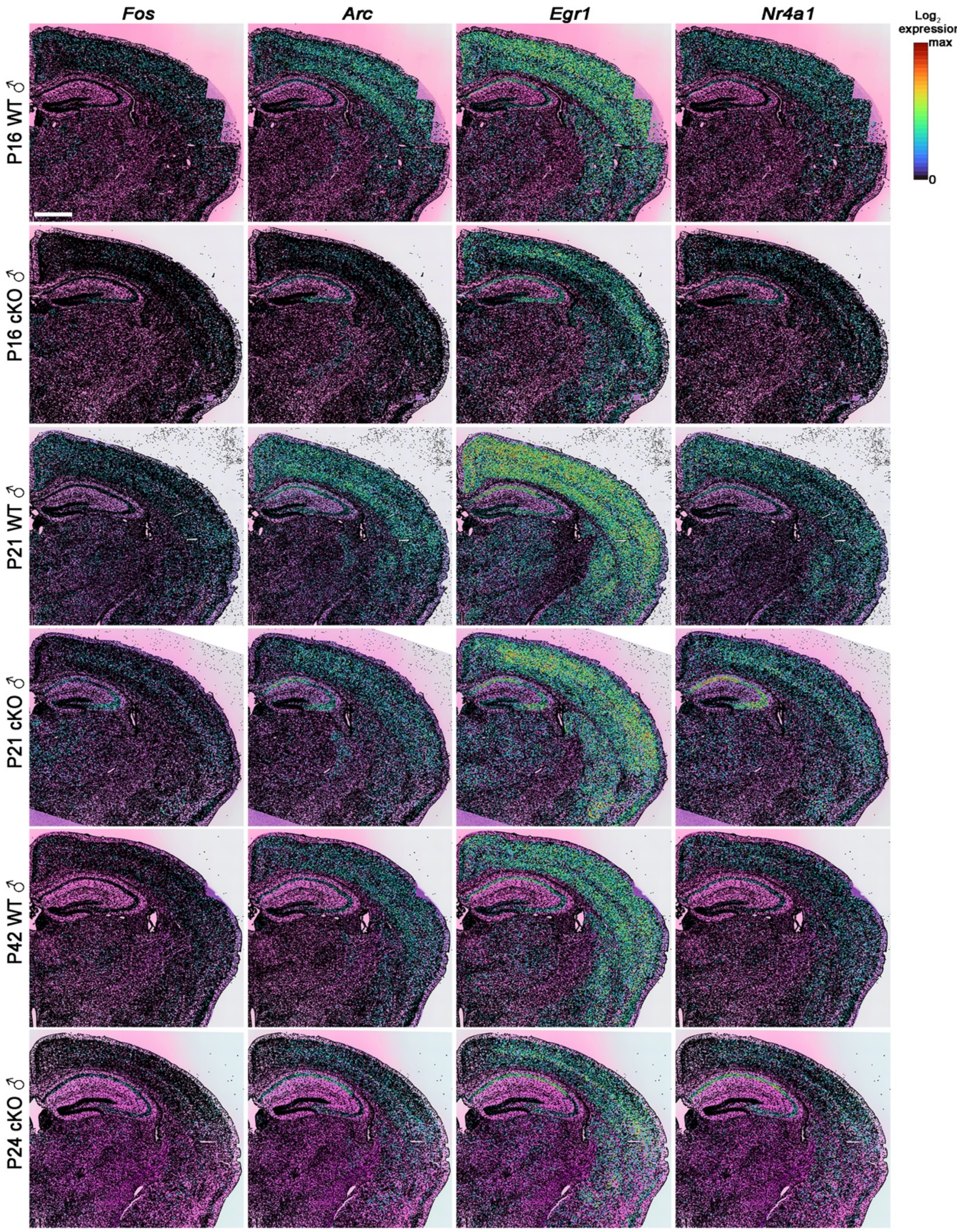

Figure S12. Spatial distribution of activity related genes in WT and cKO male postnatal brains. Spatial distribution of mRNA in P16, P21 and P42 WT and cKO male mice for *Fos*, *Arc*, *Egr1* and *Nr4a1* visualized in cell segmented spatial transcriptomics data. Scale bar = 1 mm.

bioRxiv preprint doi: https://doi.org/10.64898/2026.09.08.749487; this version posted September 10, 2026. The copyright holder for this preprint (which was not certified by peer review) is the author/funder. This article is a US Government work. It is not subject to copyright under 17 USC 105 and is also made available for use under a CC0 license.

Figure S13

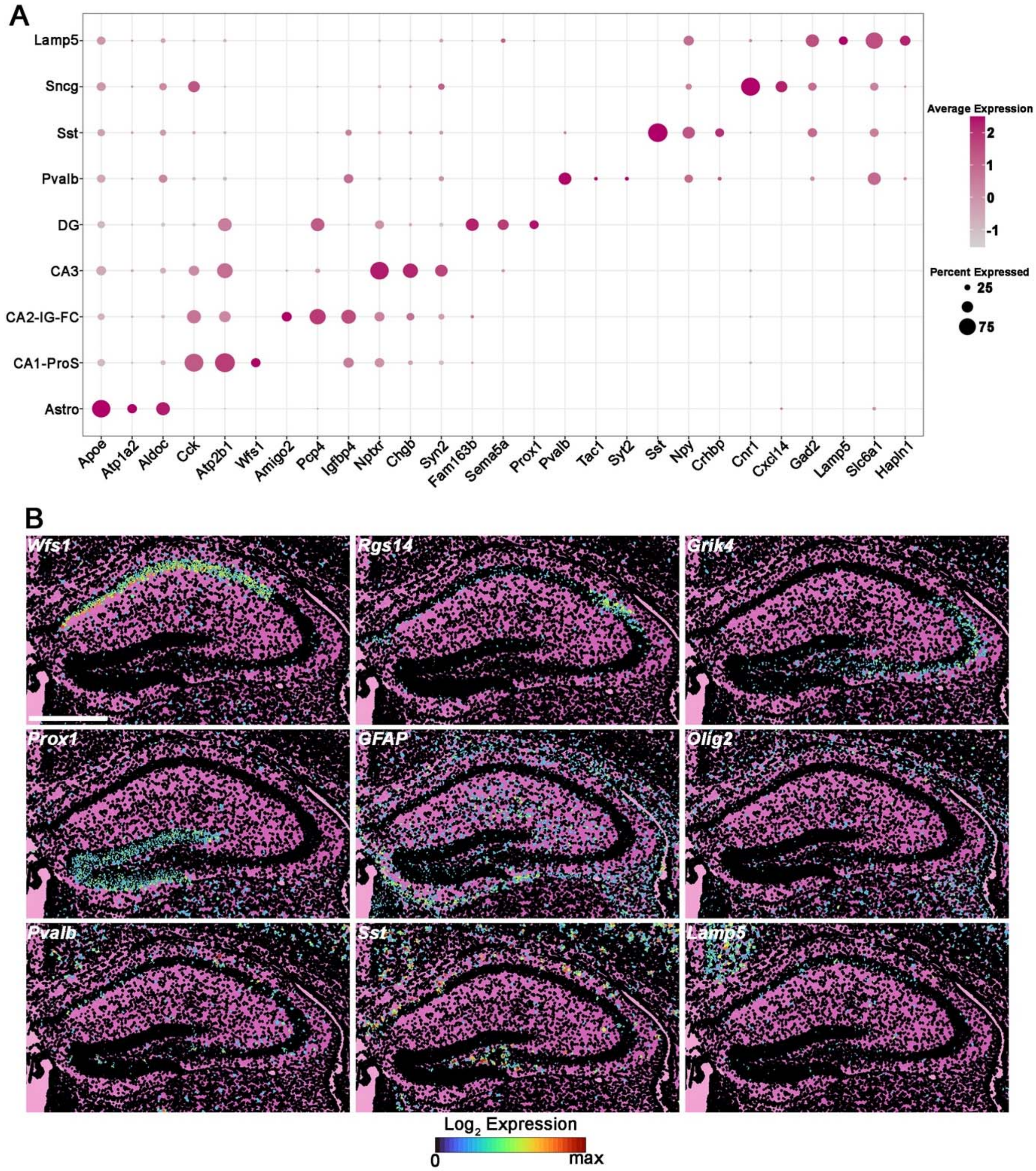

Figure S13. Genes defining hippocampal subdomains and cell types. A. Dot plot for depicting genes enriched in hippocampal subdomains and cell types. **B.** Spatial distribution of mRNA in P16 WT male hippocampus visualized in cell segmented spatial transcriptomics data. Scale bar = 0.5 mm.

Figure S14

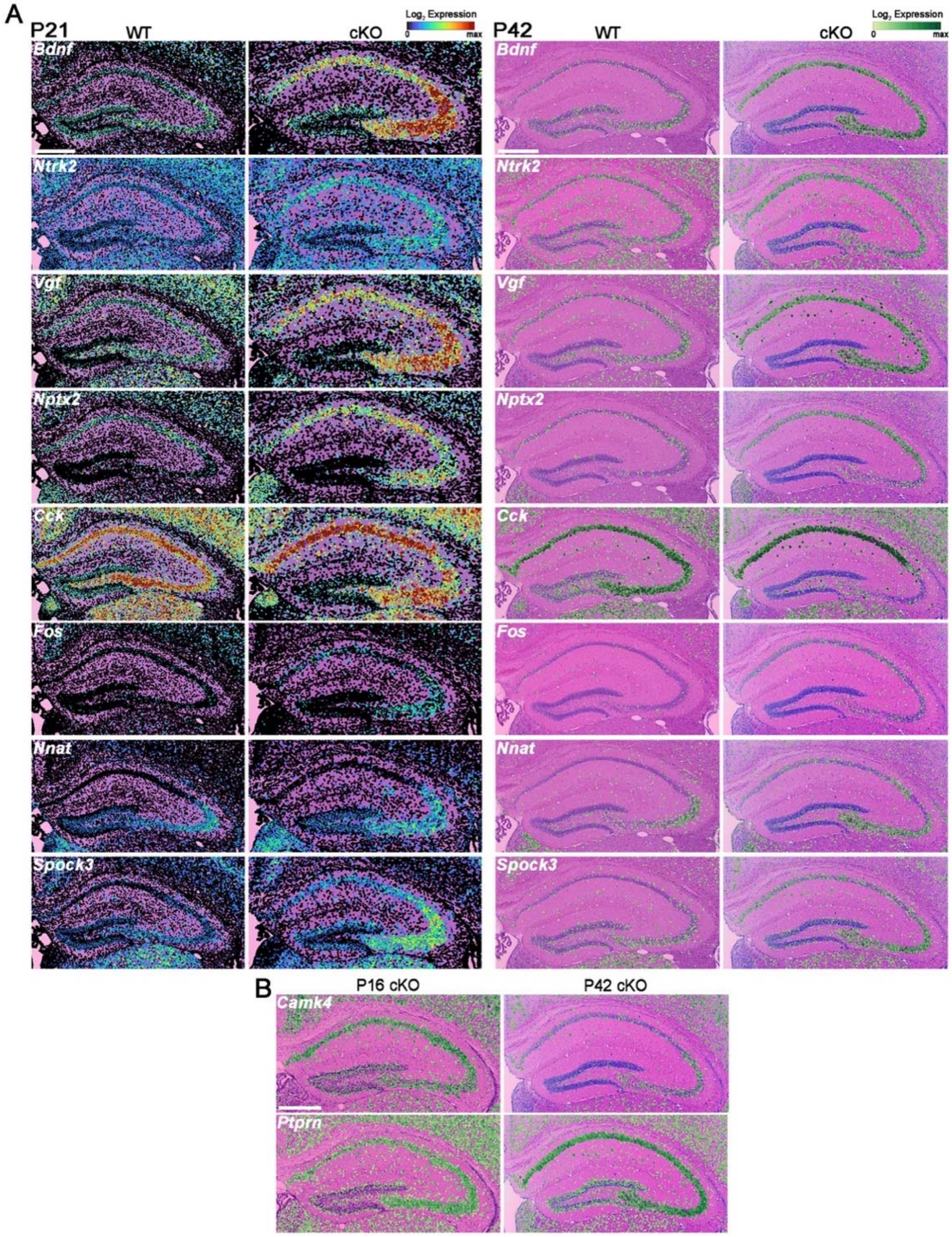

Figure S14. Spatial distribution of seizure related genes transcripts in WT and cKO male hippocampus. A. Spatial distribution of mRNA in P21 (left) and P42 (right) WT and cKO male hippocampus visualized in cell segmented spatial transcriptomics data. **B.** Spatial distribution of mRNA in P16 (left) and P42 (right) cKO male hippocampus. Scale bar = 0.5 mm.

bioRxiv preprint doi: https://doi.org/10.64898/2026.09.08.749487; this version posted September 10, 2026. The copyright holder for this preprint (which was not certified by peer review) is the author/funder. This article is a US Government work. It is not subject to copyright under 17 USC 105 and is also made available for use under a CC0 license.

Figure S15

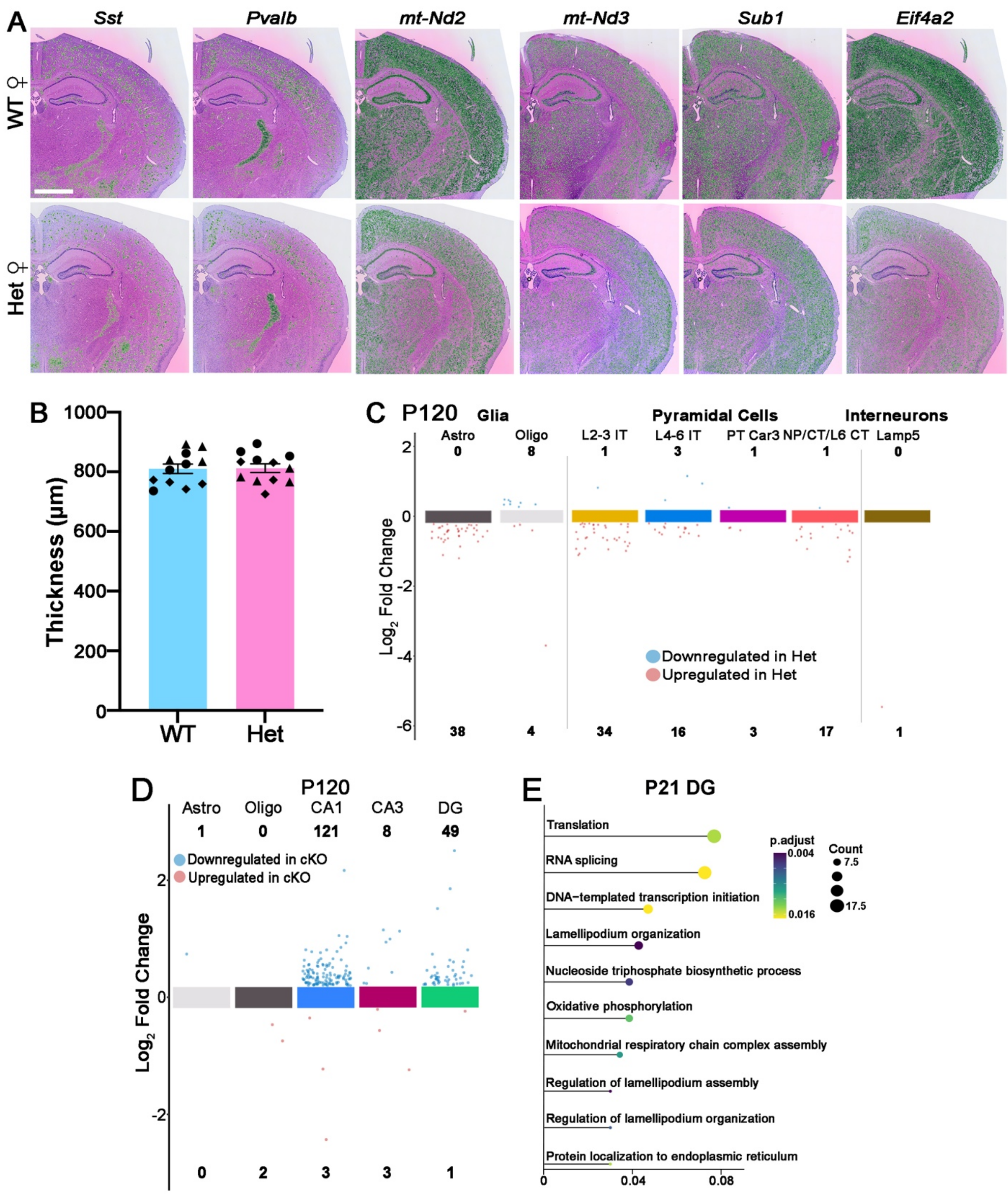

bioRxiv preprint doi: https://doi.org/10.64898/2026.09.08.749487; this version posted September 10, 2026. The copyright holder for this preprint (which was not certified by peer review) is the author/funder. This article is a US Government work. It is not subject to copyright under 17 USC 105 and is also made available for use under a CC0 license.

Figure S15. Altered gene expression in Het female mice. A. Spatial distribution of DEGs in P21 WT and Het female mice visualized in cell segmented spatial transcriptomics data. **B.** Cortical thickness in P21 female mice through S1. n = 3 brains/genotype, 12 slices counted for each genotype, each brain labeled with a different shape. **C.** Scatter chart showing DEGs downregulated (blue) or upregulated (red) in distinct cortical cell types in P120 Het mice. **D.** Scatter chart showing DEGs downregulated (blue) or upregulated (red) in Het mice in P120 hippocampus subdomains and cell types. **E.** clusterProfiler GO enrichment of top biological processes for DEGs of P21 DG cells between WT and Het mice. Welch's 2-tailed t-test (B), two-sided Wilcoxon rank sum test with Bonferroni correction (C, D), and one-sided Fisher’s exact with BH correction (E) were used. Scale bar = 1 mm.

bioRxiv preprint doi: https://doi.org/10.64898/2026.09.08.749487; this version posted September 10, 2026. The copyright holder for this preprint (which was not certified by peer review) is the author/funder. This article is a US Government work. It is not subject to copyright under 17 USC 105 and is also made available for use under a CC0 license.

1357 **Figure S16**

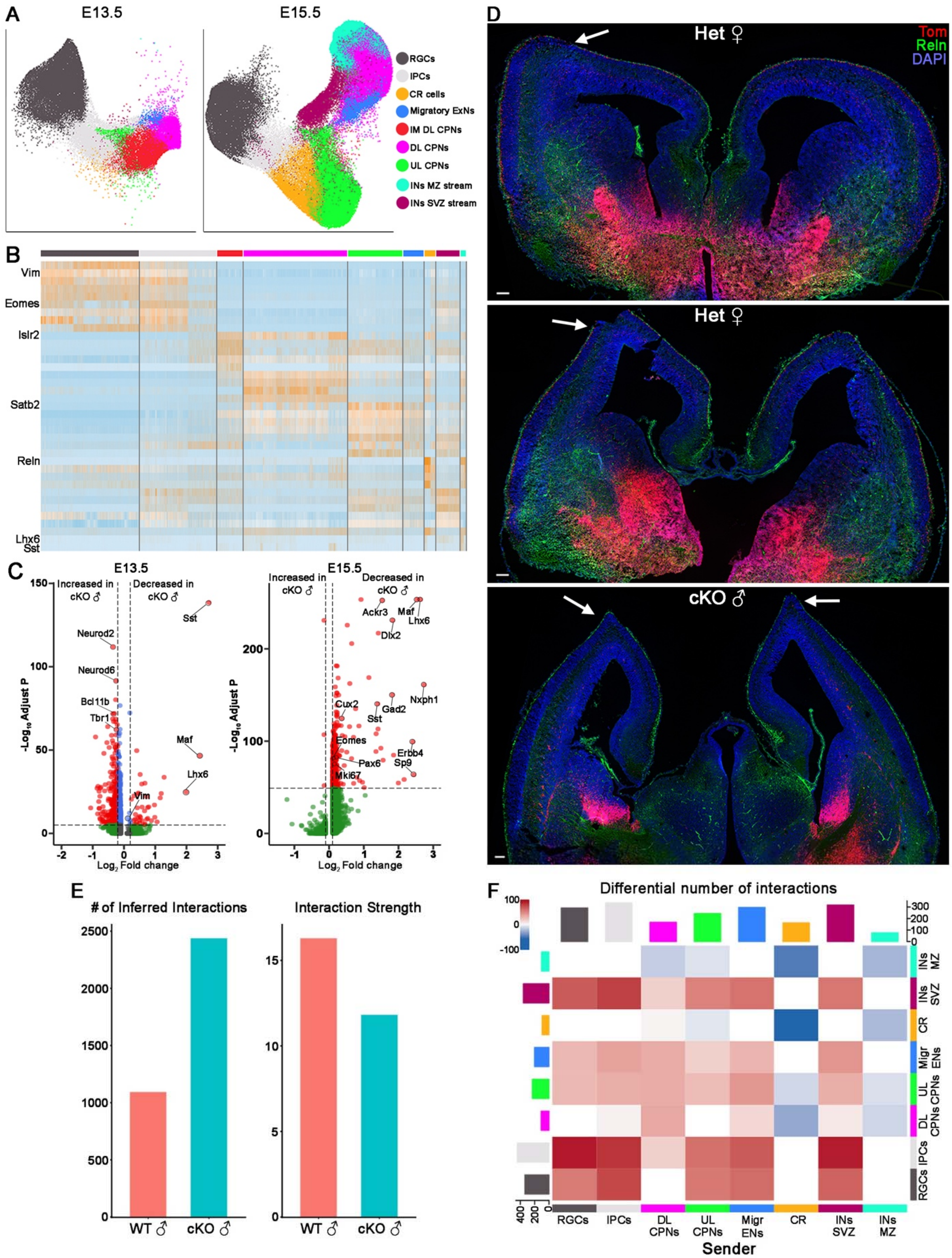

1358

bioRxiv preprint doi: https://doi.org/10.64898/2026.09.08.749487; this version posted September 10, 2026. The copyright holder for this preprint (which was not certified by peer review) is the author/funder. This article is a US Government work. It is not subject to copyright under 17 USC 105 and is also made available for use under a CC0 license.

Figure S16. Altered gene expression and cortical abnormalities in embryonic cKO mutants. A. UMAP plots of E13.5 and E15.5 cortex spatial transcripts labeled by cell types. **B.** Heatmap depicting genes enriched in embryonic cortical cell types. **C.** Volcano plot depicting E13.5 (left) and E15.5 (right) cortical DEGs between WT and cKO mice. **D.** E15.5 Het female and cKO male brain sections stained for tdTomato and Reln highlighting cortical abnormalities. Scale bar = 100 µm. **E.** Graphs depicting number of inferred interactions (left) and interaction strength (right) between E15.5 male WT and cKO cortex. **F.** CellChat-derived heatmap depicting differential number of interactions between cell types from inferred cell-cell communications in E15.5 WT and cKO cortex.
